# *Bacillus subtilis* Type I antitoxin SR6 Promotes Degradation of Toxin *yonT* mRNA and Is Required to Prevent Toxic *yoyJ* Overexpression

**DOI:** 10.3390/toxins10020074

**Published:** 2018-02-07

**Authors:** Celine Reif, Charlotte Löser, Sabine Brantl

**Affiliations:** Friedrich-Schiller-Universität Jena, Lehrstuhl für Genetik, AG Bakteriengenetik, Philosophenweg 12, D-07743 Jena, Germany; celine.reif@uni-marburg.de (C.R.); Charlotte.Loeser@uni-jena.de (C.L.)

**Keywords:** *yonT*/SR6, sRNA, small regulatory RNA, *Bacillus subtilis*, type I toxin-antitoxin system, multistress responsive TA system

## Abstract

*yonT*/SR6 is the second type I toxin-antitoxin (TA) system encoded on prophage SPβ in the *B. subtilis* chromosome. The *yonT* ORF specifying a 58 aa toxin is transcribed on a polycistronic mRNA under control of the *yonT* promoter. The antitoxin SR6 is a 100 nt antisense RNA that overlaps *yonT* at its 3′ end and the downstream gene *yoyJ* encoding a second, much weaker, toxin at its 5′ end. SR6 displays a half-life of >60 min, whereas *yonT* mRNA is less stable with a half-life of ≈8 min. SR6 is in significant excess over *yonT* mRNA except in minimal medium with glucose. It interacts with the 3′ UTR of *yonT* mRNA, thereby promoting its degradation by RNase III. By contrast, SR6 does not affect the amount or half-life of *yoyJ* mRNA. However, in its absence, a *yoyJ* overexpression plasmid could not be established in *Bacillus subtilis* suggesting that SR6 inhibits *yoyJ* translation by directly binding to its ribosome-binding site. While the amounts of both *yonT* RNA and SR6 were affected by vancomycin, manganese, heat-shock and ethanol stress as well as iron limitation, oxygen stress decreased only the amount of SR6.

## 1. Introduction

To date, six types of toxin–antitoxin systems are known (reviewed in [1,2]). All these systems comprise two genes, one encoding a stable toxin, the other an unstable antitoxin that neutralizes the toxin action. Whereas in type I and III TA systems, the antitoxin is a small RNA, in type II, IV, V and VI systems toxin and antitoxin are proteins. In the recently described type VI system, the toxin blocks replication elongation by directly binding to the β sliding clamp DnaN, whereas the antitoxin promotes toxin degradation by ClpXP [3]. Type I antitoxins interact with their toxin mRNAs that mostly code for small hydrophobic peptides recruited to the cell membrane, but also for RNA or DNA cleaving enzymes (rev. in [4]). The first TA systems have been found on plasmids, where they act as post-segregational killing (PSK) systems (rev. in [5]). Subsequently, a multitude of TA systems were discovered on bacterial chromosomes (e.g., [6]).

Type I TA systems have been found in many Gram-positive and Gram-negative species, and some of them were investigated in great detail (e.g., *hok*/Sok, rev. in [5]; or *fst*/RNAI, rev. in [7,8]). TA loci are arranged as overlapping, convergently transcribed gene pairs or as divergently transcribed pairs located apart (rev. in [4]). In the first ones, the antitoxin is a cis-encoded antisense RNA (e.g., *hok*/Sok, rev. in [9]; *txpA*/RatA [10,11]; *aapA1*/IsoA1 [12]), in the latter ones, it is a trans-encoded sRNA (e.g., *tisB*/IstR1, rev. [13]; *zor*/Orz, *ibs*/Sib and *shoB*/OhsC, rev. in [14]). In some cases, deletion of the antitoxin gene results in cell lysis [15], in others, only toxin overexpression impairs growth (*zor/*Orz, *tisB*/IstR1). Whereas TA pairs on plasmids act as PSK required for plasmid maintenance (e.g., *hok*/Sok on *E. coli* plasmid R1 or *fst*/RNAII on *E. faecalis* plasmid pAD1, rev. in [7,8]), the biological function of many chromosomal TA loci is still poorly understood. *E. coli symE*/SymR was proposed to be involved in recycling of damaged RNA generated upon SOS stress (rev. in [16]), *dinQ*/AgrB in chromosome stability [17] and *ralR*/RalA in resistance against cell-wall inhibiting antibiotics [18]. By contrast, for *E. coli tisB*/IstRI and *hokB*/SokB as well as *Streptococcus mutans fst*Sm/SrSm, a role in persister formation has been demonstrated (rev. in [4]), [19]. *B. subtilis txpA*/RatA probably helps—similar to plasmid-encoded PSK systems—to maintain the *skin* element, which is excised from the chromosome during sporulation, thereby allowing reconstitution of a functional *sigK* gene [10]. In other cases, it was speculated that toxin expression might be advantageous upon stress, as shown for some type II TA systems (rev. in [9]).

In *B. subtilis*, 14 type I TA systems were proposed [20], and five of them are located on prophage regions. Three of them—*txpA*/RatA [10,11], *bsrG*/SR4 [15] and *bsrE*/SR5 [21,22]—have been investigated in more detail. *bsrG*/SR4 located on SPβ prophage is the first discovered temperature-sensitive type I TA system, as *bsrG* RNA is rapidly degraded at 48 °C. SR4 is a bifunctional antitoxin that promotes *bsrG* RNA degradation by recruiting RNase III, and additionally induces a structural change around the *bsrG* RBS that further obstructs translation [23]. The toxin BsrG (38 aa) is recruited to the cell membrane, but does not dissipate the membrane potential. Instead, it generates membrane invaginations causing delocalization of the cell-wall synthesis machinery, which finally results in autolysis [24]. *bsrE*/SR5 is a multistress-responsive TA system encoded on the prophage-like region P6 [21]. The secondary structures of *bsrE* RNA and *bsrG* RNA are very similar, and binding between toxin mRNA and the cognate antitoxin occurs in both systems via three subsequent steps [22]. However, SR5 is—in contrast to SR4—a monofunctional antitoxin that only promotes degradation of its *bsrE* toxin mRNA by recruiting RNase III. Both *bsrE* mRNA and SR5 are rapidly degraded by RNase Y upon ethanol stress. Whereas *bsrE* RNA is influenced by temperature shock and alkaline stress, SR5 is rapidly degraded under O_2_ limitation and affected by pH stress and iron limitation [21].

Here, we confirm experimentally previous data [6,20] that *yonT*/SR6 is the second type I TA system located on the SPβ prophage of the *B. subtilis* chromosome. We identified the transcription start points of *yonT* mRNA and SR6 and analyzed the expression profiles of both RNAs in different media and under stress conditions. Furthermore, we determined the strength of the *yonT* and *sr6* promoters. In addition, we calculated the half-lives of toxin mRNA and RNA antitoxin. YonT proved to be so toxic that only a mutated *yonT* gene containing a stop instead of a start codon could be cloned in *E. coli* and established in *B. subtilis*. Determination of the intracellular concentrations of both complementary RNAs showed that SR6 is present at least at 10- to 25-fold higher concentrations than *yonT* or *yoyJ* mRNA under most growth conditions. We demonstrate by Northern blotting that SR6 acts by promoting *yonT* mRNA degradation. Furthermore, we provide indirect evidence that *yoyJ* whose RBS is complementary to the 5′ end of SR6 is a second, albeit weaker, toxin that might be also inhibited by SR6. A BLAST search for homologues of *yonT, yoyJ* and *sr6* revealed that *yonT* and *sr6* are encoded in five Bacillus species, whereas the *yoyJ* gene or its promoter was mutated, so that no functional protein can be expressed.

## 2. Results

### 2.1. yonT Is Located in an Operon, and Transcripts of Different Length Originate from p_yonT_

**Figure 1:**
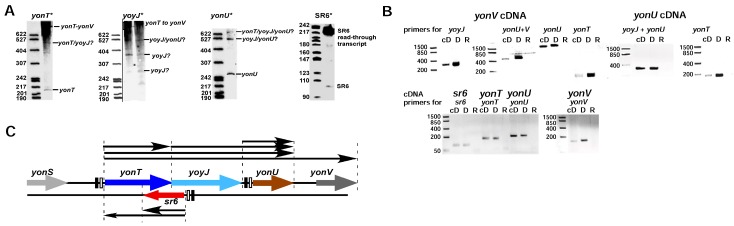
Determination of transcripts within the *yonSTUV* operon by RT-PCR. *B. subtilis* DB104 was grown in TY medium until OD_600_ = 3.0 (transition phase), total RNA isolated, treated with DNase and used as template for RT-PCR. (**A**) Northern blots. *B. subtilis* strain DB104 was grown at 37 °C in TY, aliquots taken at different times, immediately frozen in liquid nitrogen and later on used for the preparation of total RNA. RNA was separated on 6% denaturing PAA gels, blotted onto nylon membrane and hybridized with riboprobes against *yonT* mRNA, *yoyJ* mRNA, *yonU* mRNA and SR6. Autoradiograms of the corresponding gels are shown; (**B**) Original data of RT-PCR. *B. subtilis* DB104 was grown in TY medium until OD_600_ = 3.0 (transition phase), total RNA isolated, treated with DNase and used as template for RT-PCR. PCR products were analysed in ethidium-bromide stained 3% agarose gels. cD, cDNA; D, genomic DNA; R, total RNA; (**C**) Graphic summary of the transcripts in the *yonSTUV* region based on the RT-PCR data and Northern blots.

First, we performed Northern blots with total RNA isolated from *B. subtilis* wild-type strain DB104 grown in complex TY medium and probes against *yonT* RNA, *yonU* RNA and *antisense-yonT* RNA (renamed as SR6). Surprisingly, we detected *yonT* RNA species of different length from the predicted 217 over 527 to 1 kb and longer (Figure 1A) suggesting that *yonT* is located in an operon with adjacent genes. SR6 of the expected length of ≈100 nt was observed, but in addition at OD_600_ = 3.0 a longer SR6 transcript of ≈215 nt was detected. Second, to investigate if *yonT* is transcribed under an own promoter p*_yonT_* or from the upstream *yonS* promoter p*_yonS_* and is located in an operon together with the downstream genes *yoyJ, yonU* and *yonV*, we performed RT PCR with the same total RNA treated with DNase and primers for the detection of *yonT, yonU*, *yonV* and *yoyJ* transcripts. In parallel, this RNA was employed for RT-PCR with primers against SR6. Chromosomal DNA from strain DB104 served as positive control for the expected PCR fragments. Primers against *gapA* RNA were used as control for the purity and applicability of the isolated DNase-treated RNA (Figure S1). As shown in Figure 1A and supported by the RT-PCR results in Figure 1B, one long mRNA comprising *yonT, yoyJ, yonU* and *yonV* was detectable that is transcribed from promoter p*_yonT_*. Furthermore, a short 217 nt monocistronic *yonT* mRNA and a bicistronic *yoyJ/yonU* transcript—both apparently processing products of the long RNA—are visible. In addition, a tricistronic *yonT/yoyJ/yonU* transcript was found. Furthermore, a monocistronic *yonU* transcript was observed that might be transcribed from its own promoter downstream of *yoyJ* and terminated downstream of *yonU*. SR6 of the expected size could also be confirmed. An additional Northern blot with riboprobes against the upstream and downstream region of SR6 revealed that the ≈215 nt SR6 species observed at the transition to stationary growth phase at OD_600_ = 3–4 indeed originated from p*_SR6_* and extended at least towards p*_yonT_*. No signal was obtained with a probe against a sequence upstream of SR6 (not shown). Figure 1C summarizes all transcripts detected in Northern blots including the long transcript confirmed by RT-PCR in the region between p*_yonT_* and p*_yonV_*.

### 2.2. Mapping of 5′ and 3′ Ends of yonT RNA, SR6 and yonU RNA

**Figure 2:**
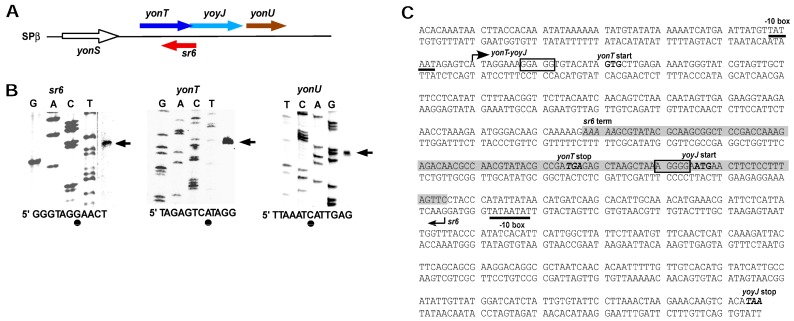
Location of the *yonT*/*sr6/yoyJ*/*yonU* locus and determination of transcription start sites. (**A**) Schematic representation of the location of the *yonT*/*sr6/yoyJ*/*yonU* locus on the *B. subtilis* chromosome. The directions of transcription are indicated by arrows; (**B**) Mapping of the 5′ ends of *yonT* mRNA, *yonU* mRNA and SR6. Sanger sequencing reactions of heterologous genes were used as references to calculate the location of the transcription start signals. Outer right lanes show primer extension reactions on total RNA of *B. subtilis* DB104 with 5′-labelled primer SB2648 (*sr6*), SB2646 (*yonT)*, and SB2647 (*yonU*) loaded in parallel to the sequencing reactions (transcription start sites are indicated by arrows). Below: *sr6, yonT* and *yonU* sequences around the TSS are shown. Dots symbolize the mapped TSS; (**C**) Sequence of the *yonT*/*yoyJ/sr6* locus. −10 boxes of the *yonT* and the *sr6* promoters are underlined. Start and direction of transcription are indicated by arrows and transcription termination is indicated by ‘term’. The *yonT*/SR6/*yoyJ* complementary region is highlighted in light grey. Start and stop codons of the *yonT* and *yoyJ* ORFs are in bold and the putative SD sequences are boxed.

An sRNA antisense to *yonT* was first detected by Fozo et al. [6]. To conform to the other known *B. subtilis* TA systems [20], we renamed antisense-*yonT* as SR6 (see above) and determined the transcriptional start sites of *yonT* mRNA, SR6 and *yonU* mRNA. The latter was included to confirm that *yonU* is transcribed from an own promoter (−10 box: TATAAT 120 bp upstream of the TTG start codon) as depicted in Figure 1C. Figure 2A presents the chromosomal location of the *yon* region and Figure 2B the results of the 5′ end mapping. No typical rho-independent terminators were found downstream of *yonT, yoyJ* and *yonU*, indicating that the monocistronic *yonT, yonU* and *yoyJ* transcripts are most likely processing products. As S1 mapping also did not allow the exact determination of the 3′ ends, the RNA sizes calculated by Northern blotting (see Figure 1A and below) were used to narrow down the 3′ ends of monocistronic *yonT* RNA, monocistronic *yonU* RNA and SR6. All three RNAs are transcribed from σ^A^ promoters, and p*_sr6_* and p*_yonT_* have extended −10 boxes. SR6 and *yonT* overlap by ≈65 nt at their 3′ ends, whereas *sr6* and *yoyJ* overlap by ≈30 to 40 nt at their 5′ ends. Figure 2C displays the complete nucleotide sequence of the *yonT/sr6/yoyJ* locus. 

### 2.3. Expression Profiles of yonT mRNA, yoyJ mRNA, yonU mRNA and SR6 in Complex TY and Minimal CSE Medium

**Figure 3:**
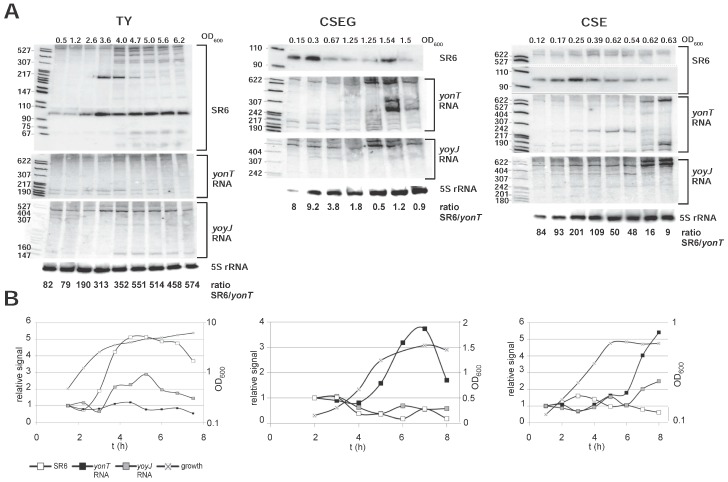
Expression profiles and concentrations of *yonT* mRNA, *yoyJ* mRNA and SR6 in complex TY and minimal CSE medium. (**A**) Northern blots. *B. subtilis* strain DB104 was grown at 37 °C in TY or CSE medium, and RNA isolation, Northern blotting and reprobing performed as in Figure 1. For the correction of loading errors, filters were reprobed with [γ-^32^P] ATP-labeled oligonucleotide C767 specific for 5S rRNA. Autoradiograms of the corresponding gels are shown. The SR6/*yonT* RNA ratios shown below the Northern blots are derived from the calculations represented by graphs in (**B**). Graphic representation of the amounts of *yonT* RNA, *yoyJ* RNA and SR6 based on the quantification of the gels shown in (**A**) and Figure S2. The corresponding growth curves are shown using the Y axis at the right side of each graph.

The expression patterns of *yonT* RNA, *yoyJ* RNA and SR6 were analyzed by Northern blotting after growth of *B. subtilis* strain DB104 at 37 °C in either complex TY or minimal CSE medium with glucose (CSEG) or without glucose (CSE). The Northern blots are displayed in Figure 3A and graphs for the calculation of RNA amounts in Figure 3B. To determine the ratio between *yonT* toxin mRNA and antitoxin SR6, defined amounts of in vitro synthesized *yonT* RNA and SR6 were loaded onto the same gel and hybridized with the same riboprobes (entire blots are shown in Figure S2). The calculations of the SR6/*yonT* RNA ratios over growth in all three media are summarized in Figure 3A below the autoradiograms of the Northern blots.

In TY medium, the amount of *yonT* mRNA was nearly unchanged over time. By contrast, the amount of *yoyJ* mRNA was two- to threefold higher in stationary phase. The amount of the antitoxin SR6 increased 5-fold from early log until transition to stationary growth phase and then slightly decreased in stationary phase (Figure 3A). Here, the SR6/*yonT* RNA ratio increased from 82 in log phase to 313 in transition phase and was 574 in late stationary phase indicating that under these conditions, toxin expression is strongly inhibited. By contrast, in CSEG medium, the amount of *yonT* RNA increased about four- to five-fold until stationary phase, whereas the amount of SR6 decreased at the same time about four- to five-fold, resulting in lowest SR6/*yonT* RNA ratios between 0.46 and 1.22 in stationary phase. This indicates that from transition phase until late stationary phase, *yonT* might be expressed in some cells. The amount of *yoyJ* RNA was nearly constant over growth. In CSE medium, the amount of SR6 was more or less constant over growth, whereas the amount of *yonT* mRNA increased in stationary phase nearly six-fold. Therefore, the SR6/*yonT* mRNA ratio decreased from logarithmic to stationary phase from 80–100 to ≈ 9, but was always in a range that would not allow toxin expression. The amounts of *yoyJ* RNA increased about two-fold towards stationary phase.

In summary, we can conclude that the amount of SR6 increased only in TY medium significantly towards stationary phase, whereas it was nearly constant in CSE. As, however, the amount of *yonT* RNA displayed a 4 or 5.5-fold increase in CSEG and CSE, respectively, the SR6/*yonT* ratio decreased in these media. But only in CSEG this ratio fell to about 1, so that only in this medium toxin expression might be expected.

### 2.4. The sr6 Promoter Is Weaker than the yonT Promoter

**Figure 4:**
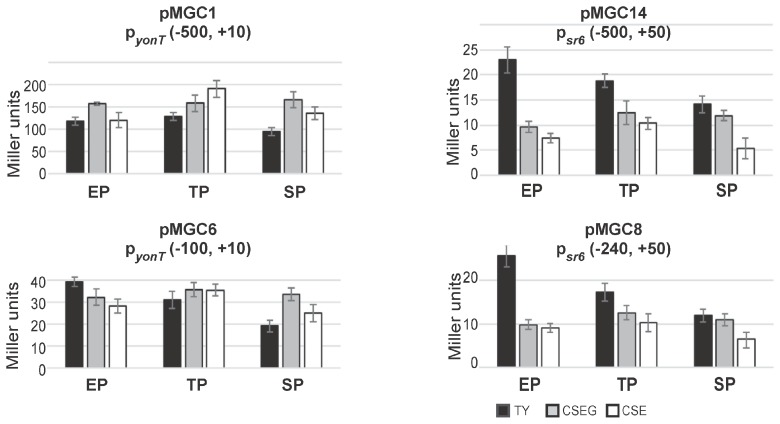
Activities of the *yonT* promoter and the *sr6* promoter in TY (black), CSEG (grey) and CSE (white) medium over growth. *B. subtilis* strains generated by integration of the indicated vector fragments into the *amyE* locus of DB104 were grown in TY, CSEG or CSE medium. In early exponential phase (EP), late exponential phase/transition phase (TP) or stationary phase (SP) samples were withdrawn and used for β-galactosidase measurements. Averages of three independent measurements with standard deviations are shown. Vector pMG16 without a promoter insert yielded in all tested media values between 1 and 3 Miller units.

To analyze activities of the promoters p*_yonT_* and p*_sr6_*, β-galactosidase measurements with transcriptional *lacZ* fusions were performed in TY, CSE and CSEG in early and late logarithmic and in stationary growth phase (Figure 4). To this end, transcriptional *lacZ* fusions comprising 500 bp upstream of the -35 boxes of p*_yonT_* (plasmid pMGC1) and p*_sr6_* (pMGC14) integrated into the *amyE* locus of strain DB104. Two additional plasmids, pMGC6 and pMGC8 carrying only 100 or 240 bp upstream of the -35 boxes, were also constructed.

As shown in Figure 4, between media and growth phases, no significant differences in the activities of p*_yonT_* could be found. The p*_yonT_*-*lacZ* fusion with the 500 bp promoter upstream region revealed in all three growth phases activities between 100 and 200 MU. In TY and CSEG medium, only a slight alteration during growth was observed, whereas in CSE medium a two-fold increase only in late exponential phase was determined. By contrast, the p*_yonT_*-*lacZ* fusion with the short (100 bp) promoter upstream region (pMGC6) revealed with 20–40 MU four- to five-fold lower activities. In TY, activities decreased from exponential to stationary phase about 2-fold, whereas in CSEG and CSE no significant alterations were observed. The activities of p*_sr6_* were with 15 to 25 MU very low. This was unexpected, as type I antitoxins are usually transcribed from rather strong promoters (e.g., a p*_sr4_*-*lacZ* fusion yielded between 750 and 3000 MU [15]). However, measurements with an empty vector yielding 0.5 MU (early log phase) to 3 MU (stationary phase) confirmed that p*_sr6_* was not inactive. The constructs pMGC14 (−500, +50) and pMGC8 (−240, +50) displayed in TY a two-fold activity decrease from early log to stationary phase, whereas in CSE and CSEG only marginal changes were observed. Similar low activities (15–27 MU) were measured for p*_yonU_* in all growth phases and media (not shown).

In summary, the measured activities of p*_sr6_* were in all growth media and stages between 20- and two-fold lower than those of p*_yonT_*. The activities of p*_yonT_* were four- to five-fold higher in constructs with a 500 bp promoter upstream region compared to those with only a 100 bp promoter upstream region suggesting a possible regulation by a transcriptional activator. Whereas p*_yonT_* activities were essentially independent of the growth medium, p*_sr6_* activities were—at least in early log phase—highest in TY medium.

### 2.5. Overexpression of yoyJ Impairs the Establishment of Transformants in B. subtilis Indicating That It Is Also a Toxin

**Figure 5:**
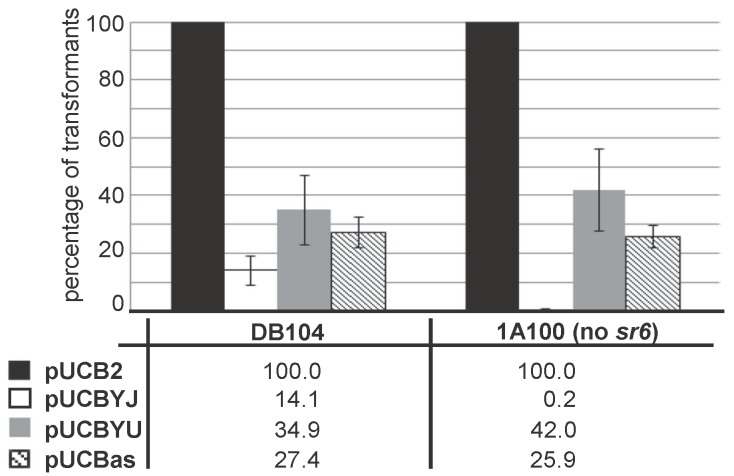
Establishment of transformants overexpressing *yoyJ* in *B. subtilis* in the presence and absence of *sr6*. *B. subtilis* strains DB104 (wild-type) and 1A100 (ΔSPβ, i.e., Δ[*yonT/yonY/sr6*]) were transformed with 5 μg purified plasmids pUCB2 (empty vector control), pUCBYJ (overexpression of *yoyJ*), pUCBYU (overexpression of *yonU*) and pUCBas (overexpression of *sr6*), transformants selected for kanamycin resistance and counted. The number of pUCB2 transformants was set to 100%. Shown data are the average of four independent experiments.

As SR6 is not only complementary to the 3′ region of *yonT* mRNA, but also to the 5′ region including the RBS of *yoyJ* (see Figure 2A,C), it was previously hypothesized [20] that YoyJ might be also a toxin, and its expression inhibited by SR6. To investigate this issue, we constructed pUCBYJ, a multicopy *E. coli/B. subtilis* shuttle vector (≈50 copies/cell) that allows for overexpression of *yoyJ* in both *E. coli* and *B. subtilis*. Already upon transformation of *E. coli* we observed rather small transformant colonies that grew much slower than those of pUCBas used for overexpression of the complete *sr6* gene. Therefore, we decided to compare the transformation efficiency of pUCBYJ in *B. subtilis* with that of pUCBas (encoding *sr6*) and pUCBYU encoding *yonU* that is not proposed to be a toxin. As control served the empty vector pUCB2. In parallel we used *B. subtilis* strain 1A100 lacking the prophage SPβ that does not encode *yonT/yoyJ/sr6* and DB104 containing SPβ, and, thus, expressing the entire *yonSTUV* operon including *sr6*. As shown in Figure 5, the transformation efficiencies of pUCBas and pUCBYU were comparable in both strains, while the empty vector pUCB2 yielded about 5 times more colonies. By contrast, pUCBYJ could not be established in strain 1A100 that does not express the antitoxin SR6, whereas it yielded a few transformants in DB104. When we grew three DB104(pUCBYJ) transformants alongside three DB104(pUCBas) transformants, they did not display altered growth (not shown). Initially we suspected that they might have undergone mutations in the *yoyJ* gene, but sequencing of 10 clones demonstrated that this was not the case.

In summary, we can conclude that YoyJ is, in addition to YonT, most probably also a toxin, although its toxicity is much lower than that of YonT. 

### 2.6. SR6 Is Significantly More Stable than yonT mRNA

**Figure 6:**
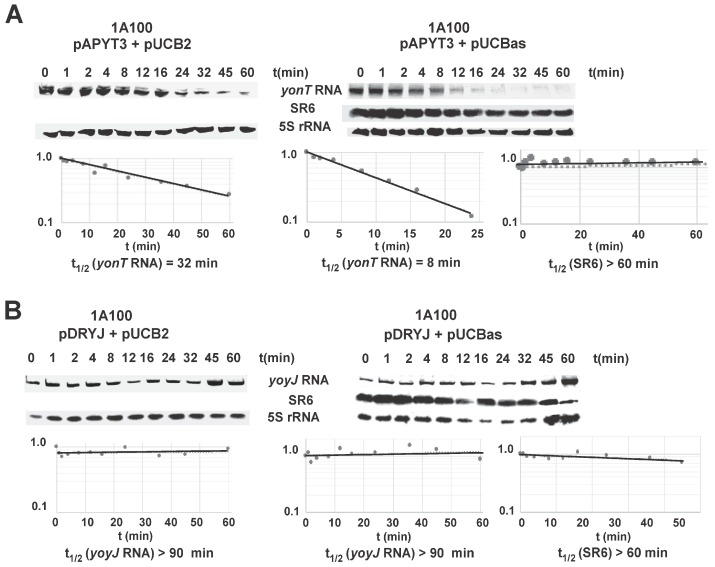
Determination of the half-lives of *yonT* mRNA and SR6 (100 nt) in complex TY medium. Half-lives were determined as described (Materials and Methods). Samples were taken at the indicated times after addition of 200 μg/mL rifampicin at OD_600_ = 3.5. [α-^32^P]-UTP-labeled riboprobes for *yonT* RNA and SR6 were used. Reprobing was performed as in Figure 3. Autoradiograms of the Northern blots are shown. Indicated half-lives are averaged from three independent determinations. (**A**) Determination of half-lives of *yonT* RNA expressed from overexpression plasmid pAPYT3; (**B**) Determination of half-lives of *yoyJ* RNA expressed from overexpression plasmid pDRYJ1. Strain 1A100 (no *sr6* gene) containing either pAPYT3 or pDRYJ1 combined with either pUCB2 (vector pUCB2) or pUCBas (pUCB2 with *sr6* gene under own promoter) were grown in TY with IPTG to induce *yonT* and *yoyJ* transcription, respectively.

Half-lives of *yonT* RNA and SR6 were determined in *B. subtilis* 1A100 in TY at OD_600_ = 3.0, i.e., at transition to stationary phase when both long and short SR6 species were visible. A *yonT* overexpression plasmid was used, as the signals from the chromosomal *yonT* gene in Northern blots were extremely weak (see Figure 3) impeding an exact calculation of a progressive decrease over time. The *yonT* RNA proved to be unstable with a half-life of 8 min (Figure 6A). The half-life of the 100 nt SR6 species was ≈60 min, whereas the 215 nt SR6 species was extremely stable with a half-life of >120 min. 

### 2.7. SR6 Promotes Degradation of yonT RNA, but Does Not Affect the Stability of yoyJ RNA

In many type I TA systems, the antitoxin promotes degradation of the toxin mRNA. To determine whether SR6 affects stability of *yonT* mRNA, SR6 was expressed in strain 1A100 from multicopy plasmid pUCBas and the empty vector pUCB2 served as negative control. The toxin gene *yonT* was expressed in the same strain from the chromosome using an integration vector (pAPYT3). Since we could not clone wild-type *yonT* in any tightly regulated inducible, high or low copy vector in *E. coli* (which is the prerequisite for analyzing *yonT* expression in *B. subtilis* 1A100)—even in the presence of a plasmid expressing *sr6*—we had to resort to cloning of a *yonT* gene with a start- to stop codon mutation (pAPYT3). In the absence of SR6, the half-life of *yonT* mRNA was ≈32 min, and it decreased to ≈8 min in the presence of SR6 (Figure 6A). With a half-life of >60 min, the stability of SR6 was not affected even by overexpression of *yonT* mRNA. This was not unexpected as we determined a high (300–350-fold in transition phase) excess of SR6 over *yonT* RNA during growth in TY medium (Figure 3A). As SR6 might also affect the half-life of *yoyJ* mRNA encoding the second toxin (see above), wild-type *yoyJ* was expressed from an integration vector (pDRYJ) in strain 1A100 either in the presence (pUCBas) or absence (pUCB2) of SR6. In both cases, *yoyJ* RNA was very stable with a half-life of >90 min, whereas the half-life of SR6 was not affected by *yoyJ* mRNA (Figure 6B).

Therefore, we can conclude that SR6 promotes degradation of *yonT* mRNA whereas it does not affect the stability of *yoyJ* mRNA.

### 2.8. The Amounts of yonT RNA and SR6 Are Affected by Different Stress Conditions

**Figure 7:**
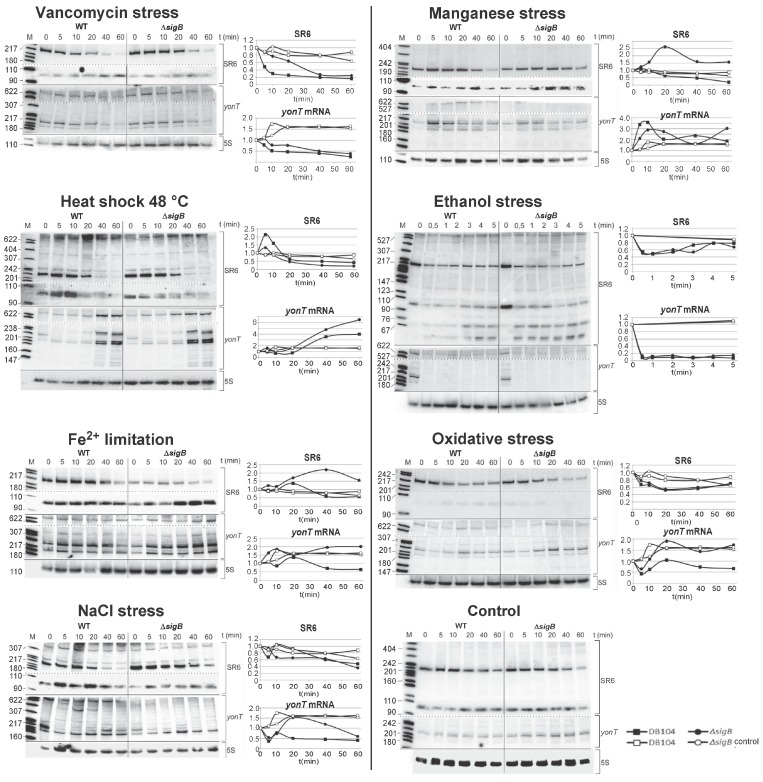
Stress conditions that influence the amounts of *yonT* RNA and SR6. *B. subtilis* strain DB104 was cultivated in TY at 37 °C until onset of stationary phase, stress factors applied as described in Materials and Methods, time samples withdrawn after indicated times, RNA prepared and subjected to Northern blotting as in Figure 1. Wild-type and Δ*sigB* strains were compared. Analysis of cell wall (vancomycin) stress, heat shock, Fe^2+^ limitation, salt, manganese, ethanol stress, and oxygen stress. Furthermore, a control blot without applied stress factors at 37 °C is included. Next to each Northern blot, the graphic representation of the stress effects on *yonT* RNA and SR6 are displayed. The Y axis refers to the relative amount of the corresponding RNA. The results of two or three independent experiments are shown.

To identify factors that might impact *yonT* or *sr6* expression, DB104 was cultivated until onset of stationary phase in TY medium, different stress conditions were applied, time samples taken over 60 min, total RNA prepared and subjected to Northern blotting (Figure 7). To find out if the stress effects depend on the alternative sigma factor *σ^B^*, a *sigB* knockout strain was used in parallel. The following stress conditions, which did not impair cell viability, were tested as described in Materials and Methods: Cell wall stress (vancomycin), manganese stress, heat-shock, ethanol stress, iron limitation, oxidative and salt stress as well as anaerobic stress with nitrate respiration. The latter stress had no pronounced effects on either RNA (not shown). The effects of the other stress conditions are discussed below and summarized in Figure 7.

The strongest effect was observed upon addition of ethanol. Within 0.5 min, *yonT* mRNA disappeared completely. By contrast, the amount of SR6 was only two-fold reduced after 1 min and increased to 80% of its initial amount after 5 min. In both cases, the effects were *sigB* independent.

Addition of vancomycin (cell-wall stress) resulted in a five-fold decrease in the amount of SR6 after 20 min whereas the amount of *yonT* RNA decreased four-fold after 60 min. Both effects were *sigB* independent. 

After heat shock (from 37 °C to 48 °C), a four-fold increase in the amount of *yonT* mRNA was observed whereas the amount of SR6 decreased four-fold after 60 min, both *sigB* independently. This is in stark contrast to the heat-shock effects on *bsrG* and *bsrE* toxin mRNAs, whose amounts both decreased 10-fold while the amounts of the cognate antitoxins SR4 and SR5 were unaffected [15,21].

Upon manganese stress, the amount of SR6 decreased four-fold after 60 min, whereas the amount of *yonT* RNA increased 3.5-fold after 10 min followed by a decrease after 20 min to the duplicate of the initial amount, both independent of *sigB*.

Lower effects were found upon iron limitation (two-fold decreased amounts of both SR6 and *yonT* RNA), oxidative stress (two-fold decrease of SR6, no significant effect on *yonT* RNA), and salt stress (two-fold decrease of *yonT* RNA, at the most two-fold effect on SR6). Interestingly, oxidative stress and iron limitation were the only stress conditions that depended on *sigB*.

Taken together, we can conclude that *yonT*/SR6 is a multistress-responsive type I TA system.

### 2.9. Homologues of the yonT, yoyJ and sr6 Are Present in Some Other Bacillus Species

**Figure 8:**
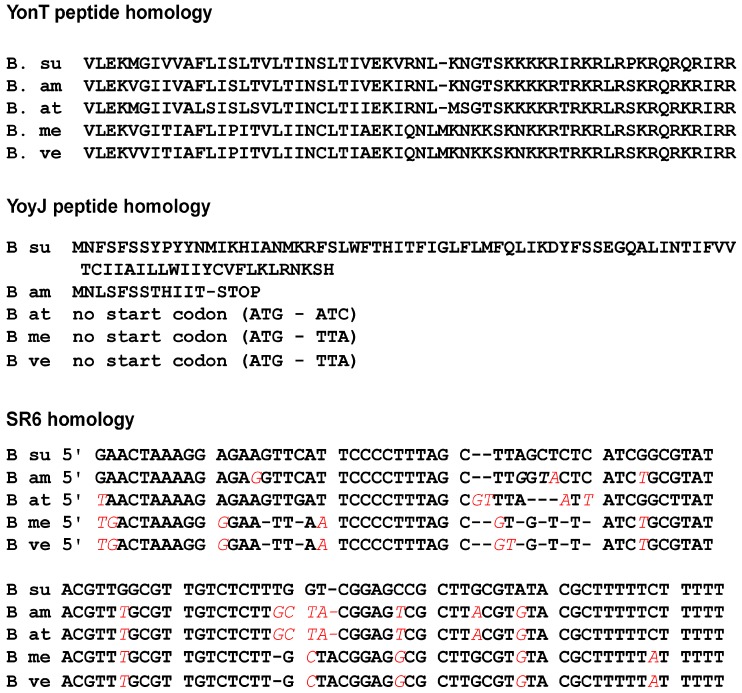
Alignments of the *sr6* DNA sequences and the YonT and YoyJ ORFs from different *Bacillus* species. BLASTn and BLASTp were applied. **Above**: Amino acid sequence alignments. Above *B. subtilis* sequence, below sequences from *B. amyloliquefaciens*, *B. athrophaeus, B. methylotrophicus* and *B. velezensis*. **Below**: DNA sequence alignment.

An inspection of all currently published genomes for homologues to *yonT, yoyJ* and *sr6* revealed DNA sequences of high similarity in *Bacillus subtilis*, *Bacillus amyloliquefaciens* XH7, *Bacillus atrophaeus* NRS 1221A, *Bacillus methylotrophicus* JJ-D34 and *Bacillus velezensis* CC09 (Figure 8). The *yonT* DNA sequence and the YonT aa sequence were highly conserved in all five strains, the latter displaying only a few aa exchanges. In addition, a protein BLAST revealed a highly similar YonT protein comprising 66 aa in *B. nakamurai* and a shortened, 37 aa homologue lacking the YonT C terminus in *B. vallismortis*. The *sr6* gene was also highly conserved in the five species mentioned first. In all cases, an extended −10 box of p*_sr6_* was detectable and only the first transcribed nt showed a few variations. By contrast, only *B. subtilis* does encode the complete *yoyJ* ORF. In *B. atrophaeus*, *B. methylotrophicus* and *B. velezensis*, the ATG start codon and the following 11 codons of *yoyJ* are missing and the shortened ORF starting with the second ATG is not preceded by a RBS, while the *B. amyloliquefaciens yoyJ* ORF carries a premature stop codon after codon 12 making it in all these cases unlikely that a functional YoyJ protein is synthesized.

## 3. Discussion

### 3.1. yonT/SR6 Is a Type I TA System That Encodes Two Toxins Regulated by Different Mechanisms

In this study, we report on the second type I TA system from the *B. subtilis* chromosome that is encoded on the SPβ prophage. In contrast to the other type I TA systems investigated so far in *B. subtilis*, but also in other bacteria, it encodes one RNA antitoxin (SR6) that regulates two toxin genes, *yonT* and *yoyJ*, employing two different mechanisms. The 3′ end of the *sr6* gene overlaps the 3′ end of the *yonT* gene by about 65 bp. Both RNAs interact by complementary base-pairing leading to the decay of *yonT* mRNA indicated by its 4-fold shorter half-life in the presence of SR6 (Figure 6A). RNase III is involved in this process: Durand et al. have shown that RNase III is in *B. subtilis*—in contrast to other bacterial species—essential because it is needed to protect this bacterium from the expression of two toxin genes, *txpA* and *yonT* encoded on two prophage-born type I TA systems, *txpA*/RatA on the skin prophage and *yonT/sr6* on the SPβ prophage [11]. Only in the presence of RNase III, the cognate antitoxins RatA and antisense-yonT (which we renamed as SR6) can prevent toxin synthesis [11]. Once both gene pairs are deleted, RNase III is not essential anymore. The mechanism of action of SR6 on *yonT* mRNA is similar to that of SR4 on *bsrG* and SR5 on *bsrE* mRNA [15,21]. In both of these cases, antitoxin binding to toxin mRNA recruits RNase III to cleave the duplex, in *bsrG* RNA at nt 14 from the 3′ end, in *bsrE* RNA 7-9 nt from the 3′ end. In contrast to SR5, SR4 is a bifunctional antitoxin as it does not only promote *bsrG* mRNA degradation but also impedes its translation by inducing a structural change that obscures the *bsrG* RBS [23]. As with translational *yonT*-*lacZ* fusions, no differences in translational activities could be observed in the absence or presence of SR6 (Table S1), a translation inhibition of *yonT* by SR6 is improbable. However, the measured β-galactosidase activities were only slightly above the negative control. Therefore, other experiments are required to corroborate these results.

Whereas SR6 acts like SR4 and SR5 by binding to the 3′ end of the toxin *yonT* RNA to promote its degradation, it overlaps the 5′ region of the second toxin gene, *yoyJ*, by approximately 30–40 bp. As we demonstrate in Figure 6, SR6 does not induce degradation of *yoyJ* mRNA. Unfortunately, employing a translational *yoyJ-lacZ* fusion under control of the strong promoter pIII [25] we could only measure activities barely above the levels of the empty vector control (Table S1), both in logarithmic or early stationary phase in the presence (strain DB104) and absence (strain 1A100) of SR6. Therefore, we resorted to in vitro *yoyJ* translation to analyze a possible effect of SR6. However, no in vitro translation product of *yoyJ* could be detected. The reason is the short distance (4 nt) between SD sequence and *yoyJ* start codon that makes *yoyJ* translation extremely inefficient (see Figure 2C).

However, transformation experiments provided an indirect evidence for the toxicity of YoyJ: *B. subtilis* strain A100 devoid of the *yonT/yoyJ*/SR6 system could not be transformed by plasmid pUCBYJ for constitutive *yoyJ* expression, whereas DB104 encoding SR6 as single copy on its chromosome, could, albeit with a somewhat lower efficiency than the same vector encoding *yonU* or *sr6* itself (Figure 5). Although this data is only an indirect evidence, it shows that *yoyJ* does encode a toxin, albeit one with considerable lower toxicity than YonT. The low toxicity of YoyJ might be due to its very weak translation. For YonT it was not possible to construct an inducible overexpression *E. coli-B. subtilis* shuttle vector even with a medium copy number and a tightly controlled promoter. This was in contrast to a former publication that reported the construction of an *E. coli* plasmid for inducible overexpression of *yonT* under the pBAD promoter [6]. We assume that a mutation occurred either in the constructed plasmid or the *E. coli* strain that decreased toxicity of YonT and allowed to establish this plasmid in *E. coli.*

The growth and establishment effects suggest a slightly toxic function of the *yoyJ* gene. The antitoxin SR6 carries a complementary region to the 5′ end of *yoyJ* which is uncommon in type I TA systems of Gram-positive bacteria, but a characteristic of Gram-negative bacterial type I TA systems such as *hok*/Sok or *ibs*/Sib of *E. coli* (rev. in [4]). At the moment, the molecular mechanism by which SR6 neutralizes the toxicity of YoyJ is unknown. A BLAST search showed that *yonT* is well conserved in five *Bacillus* species, among them *B. amyloliquefaciens* and *B. atrophaeus* (Figure 8). By contrast, the *yoyJ* gene carries different mutations in other *Bacillus* species, e.g., an early stop codon in *B. amyloliquefaciens* and a mutated start codon in the other three species which indicates that YoyJ is not synthesized in other species. 

### 3.2. Antitoxin/toxin RNA Ratios and Half-Lives

In cis-encoded sense/antisense RNA systems—to which type I TA systems belong—the amount of the antisense RNA has to be 5- to 10-fold higher than that of the sense RNA to ensure proper control (rev. in [26]). This has been also confirmed by quantitative measurements for type I TA systems *bsrG*/SR4 [27] and *bsrE*/SR5 [21]. In complex TY medium, the SR4/*bsrG* RNA ratio fell below 1.0 already at late log phase and decreased further drastically in stationary phase to 0.2 [27]. Similar results were obtained for *bsrE*/SR5 in the stationary growth phase in TY medium and in minimal CSE medium [21]. Furthermore, only under two stress conditions, anaerobic and alkaline stress, the SR5/*bsrE* RNA ratio fell well below 1, thus allowing toxin expression [21]. Here, we determined SR6/*yonT* mRNA ratios between 82 and 574 in TY medium and between 9 and 201 in CSE medium without glucose (Figure 3) excluding toxin expression under these conditions. By contrast, in CSE medium with glucose, this value fell already at late logarithmic phase below 2, indicating that *yonT* might be expressed under these conditions. However, it has to be taken into consideration that *yonT* has the rare GUG start codon preceded by an extended SD sequence with 11 bp complementarity to the anti-SD of *B. subtilis* 16S rRNA, which would efficiently recruit, but slowly release ribosomes [28], thus drastically reducing the probability of toxin synthesis [20].

A hallmark of all TA systems is a stable toxin regulated by an unstable antitoxin. This was confirmed by half-life measurements in several type I TA systems that revealed short half-lives for the regulatory RNA antitoxin and long half-lives for toxin mRNAs (rev. in [4]). In *hok*/Sok, the *hok* mRNA is extremely stable with a half-life of about 30 min, whereas antitoxin Sok is short-lived with a half-life of 1 min. Similarly, *bsrG* mRNA and *bsrE* mRNA have a half-life of 16 min and 80–120 min, respectively, whereas those of their cognate antitoxins SR4 and SR5 are 3.5 min and 7–14 min [15,21]. Surprisingly, the half-live of SR6 (both species) is with >60 min extremely long, whereas that of *yonT* mRNA is with 8 min more than 7 times shorter. As the p*_sr6_* activity is extremely low (see Figure 4), such a long half-life of SR6 might be required to ensure sufficient antitoxin levels. Due to its long half-life, the identification of RNases responsible for SR6 degradation is not feasible. Interestingly, Durand et al. postulated a half-life of ≈1.3 min for the *yonT* RNA in the presence of SR6 and a half-life of SR6 of >20 min [11]. However, they used another *B. subtilis* strain which might differ in the activity of certain RNases. In addition, their *yonT* RNA signal was barely visible making this half-life determination questionable. A determination of half-lives in the absence of SR6 was not performed by these authors. The second toxin RNA regulated by SR6, *yoyJ*-mRNA, is with a half-life of >90 min also very stable (Figure 6). Such a long half-life is rather typical for a toxin mRNA. In addition, in TY medium, but not in CSE or CSEG, two SR6 species exist, one with the expected length of about 100 nt, and a longer species of ≈215 nt (see Figure 1) that resulted from read-through of the apparently inefficient transcription terminator. The latter species was only detectable at transition phase. Currently, it is unclear which factors allow read-through from p*_sr6_* only in TY medium and during a short time-frame.

### 3.3. yonT/SR6 Is a Multistress-Responsive TA System

Toxin expression has to be tightly controlled*:* For PSK systems like *hok*/Sok and *fst*/RNAII, constitutive expression of both toxin and antitoxin promoters are required (rev. in [4]). The same seems to be true for chromosome-encoded *txpA*/RatA [10]. By contrast, *B. subtilis bsrG* and *bsrE* are temperature-sensitive [15,21]: *bsrG* mRNA has a 3- to 4-fold shorter half-life at high temperatures due to refolding at heat-shock which allows RNases Y and J1 to access newly formed single-stranded regions [27]. Furthermore, for the *bsrE*/SR5 system, multiple stress conditions affected toxin and/or antitoxin expression, the most pronounced among them were anaerobic and alkaline stress that decreased the antitoxin/toxin RNA ratio 8- to 10-fold and 4- to 5-fold, respectively, as well as ethanol stress, which caused a rapid degradation of toxin RNA by RNase Y within 0.5 min [21]. *E. coli* toxin *tisB*, *symE and dinQ* promoters are preceded by LexA boxes which make them SOS inducible whereas their antitoxins are expressed constitutively (rev. in [13,16]). In the case of SymE, an additional post-translational regulation by Lon protease was reported [16]. *S. aureus sprA1* expression decreases two-fold at lower pH, while it increases three-fold under oxidative stress [29]. Interestingly, *E. coli ralR* is higher expressed in later stages of biofilm development [30]. Likewise, chromosome-encoded type II TA systems as *relBE or mazEF* were discovered to be sensitive to nutritional stress during growth (rev. in [4]).

Here, we report the effects of several stress factors on *yonT* mRNA and SR6 (see Figure 7). As in the case of *bsrE* mRNA, the strongest effect was found for ethanol: within 0.5 min after ethanol addition, *yonT* mRNA disappeared completely. In contrast to *bsrE*/SR5, oxygen deficiency did not have significant effects. However, the cell-wall toxin vancomycin, that did neither impact *bsrE* RNA nor SR5, resulted in a four- and five-fold decrease of *yonT* RNA and SR6, respectively. This would, however, not alter the SR6/*yonT* ratio making toxin expression under cell-wall stress unlikely. Interestingly, oxidative stress and iron limitation which resulted in about 2-fold effects on *yonT* RNA and SR6, were the only stress conditions dependent on *sigB*. In the case of *bsrE*/SR5, iron limitation increased the amount of antitoxin SR5, also *sigB* dependent, but oxidative stress had no effect. However, neither in *bsrE*/SR5 nor *yonT*/SR6 an additional *sigB* promoter could be detected, so that SigB dependency is most likely due to other factors controlled by this alternative sigma factor. Manganese stress had no effect on *bsrE*/SR5, but affected *yonT*/SR6 significantly, and salt stress had a small impact on *yonT*/SR6 while it exerted no effect on *bsrE*/SR5. Surprisingly, temperature shock had the reverse effect on *yonT*/SR6 compared to *bsrE*/SR5 or *bsrG*/SR4 [15,21]: It caused a four-fold increase in the amount of *yonT* mRNA whereas that of SR6 was reduced four-fold after 60 min. By contrast, *bsrG* and *bsrE* mRNA amounts decreased 10-fold after heat-shock while the amounts of both antitoxins remained constant. Based on these observations, it can be assumed that the toxin YonT might be synthesized upon heat shock. Since secondary structures of *yonT* RNA or SR6 have not been mapped so far, we currently cannot provide a mechanistic explanation for the heat-shock effects on the amounts of SR6 and *yonT* RNA.

In summary, we can conclude that *yonT*/SR6 is also a multistress-responsive type I TA system, and that most probably *yonT* is expressed upon heat-shock conditions at least in individual cells of the culture.

Future investigations will focus on the elucidation of the mechanisms responsible for the stress effects and on the cellular targets of the two toxins, YonT and YoyJ.

## 4. Materials and Methods

### 4.1. Enzymes and DNA Manipulations

Taq DNA polymerase (Roche), Firepol polymerase (Solis Biodyne), sequenase and Thermoscript reverse transcriptase (Thermoscientific), as well as T7 polymerase and polynucleotide kinase (New England Biolabs) were used. DNA manipulations like plasmid purification, *E. coli* and *B. subtilis* transformation were carried out as described [31].

### 4.2. Strains, Media and Isolation of Chromosomal DNA

*E. coli* strain DH5α and *B. subtilis* strains DB104 and 1A100 (ΔSPβ) were used (see Table 1). 1A100 was included as this strain does not carry the *yonT*/*sr6* region encoded on the SPβ prophage. TY served as complex medium for *E. coli* and *B. subtilis* and CSE as minimal medium for *B. subtilis* [32]. Chromosomal DNA from *B. subtilis* strains was isolated as described [33].

### 4.3. Primer Extension

Primer extension experiments were carried out as described [33] using total RNA from *B. subtilis* strain DB104 and 5′-labelled primer SB2646 for *yonT* RNA, SB2648 for SR6 and SB2647 for *yonU* RNA (primers are listed in Table S2).

### 4.4. Vector Construction

For the determination of promoter activities, transcriptional *lacZ* fusion plasmids were constructed as follows: A PCR was performed on chromosomal DNA of *B. subtilis* DB104 with primer pairs SB2598/2599 (pMGCR1/5), SB2628/2599 (plasmid pMGCR6), SB2630/2644 (pMGCR8), SB2600/SB264 (pMGCR14) and SB2630/SB2706 (pMGCR16). All primers are listed in Table S2, and all plasmids in Table 2. The resulting fragments were cleaved with BamHI and EcoRI—except pMGCR1/5 that was cleaved with BglII and BamHI—and inserted into the pMG16 vector digested with the same enzyme pair. Plasmids pMGCR1/5 and pMGCR6 contain p*_yonT_* from −500 and −100, respectively, to +10 with regard to the transcriptional start site (TSS) fused to the promoterless *lacZ* gene, whereas pMGCR14 comprises p*_sr6_* with −500 to +50 and pMGCR8 and pMGCR16 p*_sr6_* with −240 to +50, respectively. Translational *yonT-lacZ* (pGAY1) and *yoyJ-lacZ* (pGAY2) fusions were constructed using a PCR with primer pairs SB2760/2761 (pGAY1) or SB2764/SB2765 (pGAY2), respectively on chromosomal DNA. The resulting fragments were digested with BamHI and EcoRI and inserted into the BamH/EcoRI pGAB1 vector. In all cases, the native promoter was replaced by the strong constitutive heterologous promoter pIII [25].

To overexpress *sr6, yoyJ* and *yonU* in *Bacillus subtilis*, plasmids pUCBas, pUCBYJ and pUCBYU were constructed, respectively, as follows: PCR fragments were obtained on chromosomal DNA from *B. subtilis* strain DB104 with primer pairs SB2654/2655 (pUCBas), SB2690/2691 (pUCBYJ) or SB2635/SB2636 (pUCBYU), cleaved with BamHI and HindIII and inserted into the BamHI/HindIII vector of pUCB2. The cognate p*_sr6_* and p*_yonU_* promoters and, in case of pUCBYJ, p*_yonT_*, were used. Primer SB2636 contains the heterologous *bsrF* terminator at its 3′ end [36]. pANC213cat is a vector for integration into the alkaline phosphatase gene (*aprE* gene) that comprises the tightly controlled IPTG-inducible p_SPAC_ promoter. A promoterless *yonT* fragment generated on chromosomal DNA as template with primer pair SB2660/SB2661 was cleaved with BamHI and EcoRI and inserted into the BamHI/EcoRI pANC213cat vector. In the resulting plasmid pAPYT3, the *yonT* start codon was replaced by a stop codon and the *bsrF* terminator added at the 3′ end. A promoterless *yoyJ* fragment was produced by PCR on genomic DNA with primer pair SB2722/SB2723, digested with SphI and HindIII and cloned into the SphI/HindIII pDR111 [37] vector that contains the IPTG-inducible hyperspank promoter and can be integrated into the *B. subtilis amyE* locus.

### 4.5. β-Galactosidase Assay

The transcriptional and translational fusions were integrated into the *amyE* locus of the *B. subtilis* DB104 or 1A100 chromosome and β-galactosidase activities were determined as described [38]. 

### 4.6. Determination of the Intracellular Concentrations of yonT mRNA, yoyJ mRNA and SR6

The intracellular amounts of the RNAs were determined as described previously [21].

### 4.7. Preparation of total RNA, RNA Gel Electrophoresis and Northern Blotting

Preparation of total RNA, RNA gel electrophoresis on 6% denaturing polyacrylamide gels, and Northern blotting were carried out as described previously [33]. For the detection of *yonT* mRNA, *yoyJ* mRNA, *yonU* mRNA and SR6, [α-^32^P] UTP-labeled riboprobes were used generated with T7 RNA polymerase on PCR-derived DNA fragments as described [39]. RNA half-life determinations were performed according to [40], except that 200 μg/mL rifampicin were used.

### 4.8. Stress Conditions

The following stress conditions were applied (final concentrations are indicated): ethanol stress (4% ethanol), oxidative stress (10 mM H_2_O_2_), salt stress (0.5 M NaCl), Mn^2+^ stress (1 mM MnCl_2_), iron depletion (250 μM 2,2-Dipyridyl-N,N-dimethylsemicarbazone = DIP, dissolved in DMSO) and cell wall antibiotic stress (10 and 20 μg/mL vancomycin). For heat shock, cultures were shocked from 37 °C to 48 °C.

## Figures and Tables

**Table 1:** Bacterial strains.

Strain	Genotype	Reference
*E. coli* DH5α	*fhu2*, *Δ(argF-lacZ)*, *U169*, *phoA*, *glnV44*, *Φ80*, *Δ(lacZ)M15*, *gyrA96*, *recA1*, *relA1*, *endA1*, *thi-1*, *hsdR17*	[34]
*B. subtilis* DB104	*His*, *nprR*, *2 nprE18*, *ΔaprA3*	[35]
*B. subtilis* 1A100	168 *ΔSPβ*, *trpC2*	Ohio strain collection

**Table 2:** Plasmids used in this study.

Plasmid	Description	Reference
pUCB2	Shuttle vector of pUC19 and pUB110, Neo^R^, Phleo^R^	[38]
pDR111	Vector for IPTG-inducible overexpression and integration into the *B. subtilis amyE* locus, Spec^R^	[37]
pAPNC213cat	Vector for IPTG-inducible overexpression and integration into the *B. subtilis AP* locus, Cm^R^	[24]
pMG16	Vector for integration of transcriptional *lacZ* fusions into *B. subtilis amyE* locus, Spec^R^	M. Gimpel, unpublished
pGAB1	Vector for integration of translational *lacZ* fusions into *B. subtilis amyE* locus, Kan^R^	S. Brantl, unpublished
pMGCR1	pMG16 with *yonT* from −500 to +10 *	This study
pMGCR6	pMG16 with *yonT* from −100 to +10 *	This study
pMGCR8	pMG16 with *sr6* from −240 to +50 *	This study
pMGCR14	pMG16 with *sr6* from −500 to +50 *	This study
pGAY1	pGAB1 with *yonT* gene	This study
pGAY2	pGAB1 with *yoyJ* gene	This study
pUCBas	pUCB2 with *sr6* gene	This study
pUCBYT	pUCB2 with *yonT* gene	This study
pUCBYJ	pUCB2 with *yoyJ* gene	This study
pUCBYU	pUCB2 with *yonU* gene	This study
pAPYT3	pAPNC213cat with *yonT* gene (M1Stop)	This study
pDRYJ	pDR111 with *yoyJ* gene	This study

* with regard to the TSS (transcription start site).

## Supplementary Materials

The following are available online at http://www.mdpi.com/2072-6651/10/2/74/s1, Figure S1: RT PCR on *gapA* RNA, Figure S2: Calculation of the amounts of *yonT* RNA and SR6 using standard curves (shown above) obtained by loading and Northern blotting of defined amounts of in vitro synthesized *yonT* RNAand SR6 in the range of 0.01 to 5 fmol in parallel onto the gels with RNA samples from cultures grown in TY, CSEG and CSE medium (see Figure 3 in the main body of publication), Table S1: β-galactosidase-activities of translational *yonT-*1 *lacZ* and *yoyJ lacZ fusions*, Table S2: Oligonucleotides used in this study.

## Abbreviations

The following abbreviations are used in this manuscript:

aa, amino acid; nt, nucleotide; bp, base pair; sRNA, small RNA, RBS, ribosome binding site, TA, toxin-antitoxin.
