# Supplementary material for: Bacillus subtilis Type I antitoxin SR6 Promotes Degradation of Toxin yonT mRNA and Is Required to Prevent Toxic yoyJ Overexpression
Source: Toxins (Basel). 2018 Feb 7;10(2):74. doi: 10.3390/toxins10020074 (PMC5848175; doi:10.3390/toxins10020074)
Supplement: Supplementary file 1 [file toxins-10-00074-s001.pdf]

Supplementary Materials: *Bacillus subtilis* Type I antitoxin SR6 Promotes Degradation of Toxin *yonT* mRNA and Is Required to Prevent Toxic *yoyJ* Overexpression

Celine Reif, Charlotte Löser and Sabine Brantl

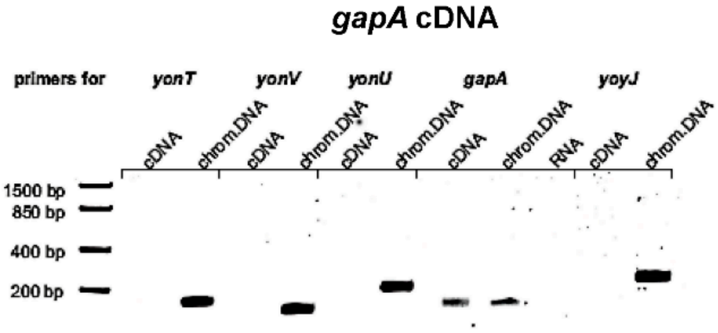

Figure S1: RT PCR on *gapA* RNA. Total RNA of *B. subtilis* DB104 grown until OD_{600} = 3.0 was isolated and used for RT PCR. Chrom. DNA = genomic DNA used as positive control. Total RNA was used as negative control.

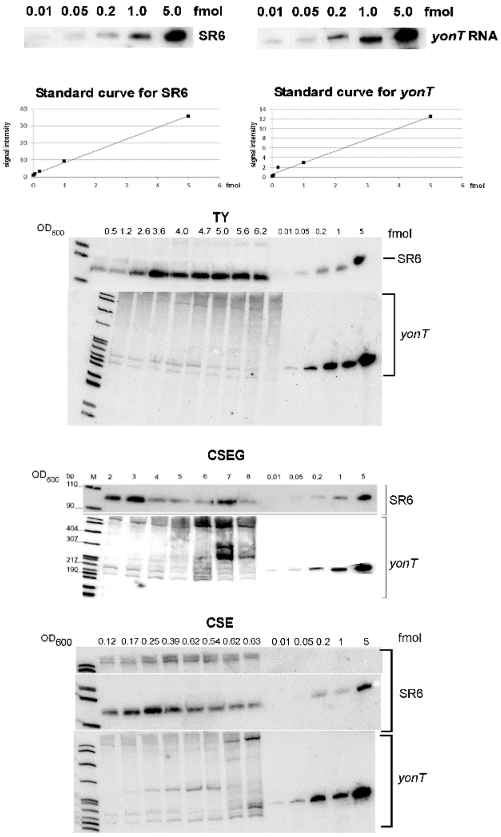

Figure S2: Calculation of the amounts of *yonT* RNA and SR6 using standard curves (shown above) obtained by loading and Northern blotting of defined amounts of *in vitro* synthesized *yonT* RNA and SR6 in the range of 0.01 to 5 fmol in parallel onto the gels with RNA samples from cultures grown in TY, CSEG and CSE medium (see Figure 3 in the main body of publication).

Table S1: β-galactosidase-activities of translational *yonT-lacZ* and *yoyJ lacZ* fusion strains.

Strain	optical density	β-galactosidase activity (MU)
DB104 (*amyE::pGAY1*)	1.0	4.3±1.4
	3.0	6.6±1.8
	5.0	10.8±2.1
DB104 (*amyE::pGAY2*)	1.0	4.1±1.1
	3.0	10.3±2.5
	5.0	10.8±1.8
DB104 (*amyE::pGAB1*)	1.0	3.1±0.7
	3.0	3.4±1.3
	5.0	7.2±1.7
1A100 (*amyE::pGAY1*)	1.0	5.3±1.1
	3.0	7.0±2.0
	5.0	13.8±1.5
1A100 (*amyE::pGAY2*)	1.0	4.1±1.1
	3.0	7.5±2.0
	5.0	11.5±1.8
1A100 (*amyE::pGAB1*)	1.0	2.9±0.9
	3.0	5.8±2.2
	5.0	8.2±2.4

B. subtilis strains were grown in TY medium, and at the indicated optical densities, samples were withdrawn and used for β-galactosidase measurements. Averages of three independent measurements with standard deviations are shown. Whereas DB104 expresses SR6 from a single-copy in the chromosome, 1A100 lacks the SPβ1 prophage and therefore, the *sr6* gene.

Table S2: Oligonucleotides used in this study.

Name	Sequence	Purpose
**Primer for sequencing**		
SB2627	5′ GTTCGTATGTATTCAAATATATCCTCC	Sequencing of pMG16 derivatives
SB1170	5′ CAGGGTTTTCCCAGTCACGAC	Sequencing of pUCB2 derivatives
SB1171	5′ GGATAACAATTTCACACAGGA	Sequencing of pUCB2 derivatives
SB1678	5′ ACGTCCGCATGCAAAAAGACCAGGGTGTTG	Sequencing of pDR111 derivatives
SB2192	5′ CCACTCAATGCCGTTAAT	Sequencing of pAPNC213cat derivatives
**Primer for reverse transcription**		

SB2649	5′ GTTGGCGTTGTCTCTTTGGTC	3′ end of *yonT*
SB2651	5′ GAACGATAATTGCCTTACCGC	3′ end of *yonU*
SB2652	5′ GTTACCTCATCAAGATATGGTG	3′ end of *yonV*
SB2711	5′ CTTGTGAATGCGTGTATCCCA	3′ end of *yonX*
SB2653	5′ CGCAAGCGGCTCCGACCAAAG	3′ end of *sr6*
	**Primer for the PCR after RT**	
SB2575	5′ GTGCTTGAGAAAATGGGTATC	5′ end of *yonT*
SB2690	5′ ATGAATTATGTTATAATAGAGTCAGAGCTAAGCTAAAGGGGAATG	5′ end of *yoyJ*
SB2650	5′ CTTAGTTTAAGGAATACACAATAGATG	3′ end of *yoyJ*
SB2579	5′ GATGCTATTCAGCAGCTAACA	5′ end of *yonU*
SB2714	5′ GGATCATCTATTGTGTATTCCTTAAAC	3′ end of *yoyJ*
SB2581	5′ TAGGAACTAAAGGAGAAGTTC	5′ end of *sr6*
SB2704	5′ AGGAGGATATATGGAAAGAGTTAA	5′ end of *yonV*
SB2712	5′ ATCTGGAGTCTAGACTTGATGAAT	5′ end of *yonX*
SB2713	5′ GCTGTTAATGGATTGGTTAGA	3′ end of *yonV*
SB1803	5′ TGCAAGGTCAACAACGCGGGCAGAGTAGCCGCTTTCGTT	3′ end of *gapA*
SB1911	5′ GGAATCCGGCTACAGCGAA	5′ end *of gapA*
	**Primers for riboprobes**	
SB2574	5′ GAAATTAATACGACTCACTATAGGCATCGGCGTATACGTTGGCGTTGT	*yonT* (up)
SB2575	5′ GTGCTTGAGAAAATGGGTATC	*yonT (*down*)*
SB2576	5′ GAAATTAATACGACTCACTATAGGAATACACAATAGATGATCCATAAC	*yoyJ* (up)
SB2577	5′ ACATGATCAAGCACATTGCAA	*yoyJ* (down)
SB2578	5′ GAAATTAATACGACTCACTATAGGTTAATTGTCCTTGTGTTGCTGCAT	*yonU* (up)
SB2579	5′ GATGCTATTCAGCAGCTAACA	*yonU* (down)
SB2580	5′ GAAATTAAACGACTCACTATAGGGCGTATACGCAAGCGGCTCCGAC	*sr6* (up)
SB2581	5′ TAGGAACTAAAGGAGAAGTTC	*sr6* (down)
SB2768	5′ GAAATTAATACGACTCACTATAGGCATGATCAAGCACATTGCAAACATG	*sr6* up (up)
SB2769	5′ GATGAGTTGAAACATTAAGAATAAGC	*sr6* up (down)
SB2766	5′ GAAATTAATACGACTCACTATAGGCGTAGTTGCTTTCCTCATATCTTTAAC	*sr6* down (up)
SB2767	5′ CCCATTCTTTAGGTTTCTTACC	*sr6* down (down)
SB767	5′ GGGTGTGACCTCTTCGCTATCGCC ACC	*5S* rRNA
	**Primers for the determination of 5′ ends**	
SB2646	5′ GAGGAAAGCAACTACGATACCC	*yonT*
SB2647	5′ CTTGAGCATTTCCAATTCCTTTG	*yonU*
SB2648	5′ GCTCCGACCAAAGAGACAACG	*sr6*
	**Primers for *in vitro* transcription**	
SB2732	5′ GAAATTAATACGACTCACTATAGGATAGGAAAGGAGGTGTACATA	*yonT*
SB2733	5′ CTTTAGCTTAGCTCTCATCGG	*yonT*
SB2734	5′ GAAATTAATACGACTCACTATAGGGAGCTAAGCTAAAGGGGAATGAA	*yoyJ*
SB2735	5′ GAGGCCTATATGATTAGTGTG	*yoyJ*
SB2736	5′ GAAATTAATACGACTCACTATAGGATTGAGGTGAGATGATTGGAG	*yonU*
SB2737	5′ AAATAAGGAACGATAATTGCCTTACCG	*yonU*
SB2738	5′ GAA ATT AAT ACG ACT CAC TAT AGG GAACTAAAGGAGAAGTTCATTCCC	*sr6*
SB2739	5′ AAAGAAAAAGCGTATACGCAAG	*sr6*
	**Primers for transcriptional and translational *lacZ* fusions**	
SB2598	5′ ATCGAATTCCTCGACGAGTACGTAGGA	pMGCR1/5
SB2599	5′ ATCGGATCCTCCTTTCCTATGACTCTATTA	pMGCR1/5
SB2631	5′ ATC GAATTC AAGGACAGGCGCTAATCAAC	pMGCR5
SB2632	5′ ATC GGATCC TTTCTCCAATCATCTCACCT	pMGCR5
SB2628	5′ ATC GAATTC TCAAACAGCACACAAGGC	pMGCR6

SB2599	5′ ATCGGATCCTCCTTTCCTATGACTCTATTA	pMGCR6
SB2630	5′ ATC GAATTC TATACCATGAGGCCTATATG	pMGCR8
SB2644	5′ ATCGGATCCACGCCGATGAGAGCTAAGCTA	pMGCR8
SB2600	5′ ATCGAATTCTCCTTGTGTTGCTGCATG	pMGCR14
SB2644	5′ ATCGGATCCACGCCGATGAGAGCTAAGCTA	pMGCR14
SB2630	5′ ATC GAATTC TATACCATGAGGCCTATATG	pMGCR16
SB2706	5′ ATCGGATCCCCGACCAAAGAGACAACGCCA	pMGCR16
SB2760	5′ GATCCCCATGGTTGAAATCCCCTCAAAAACCGATATAATGGGTTTATA GGAAAGGAGGTGTACATAGTGCTTGAGG	pGAY1
SB2761	5′ AATTCCTCAAGCACTATGTACACCTCCTTTCCTATAAACCCATTATATCG GGTTTTTGAGGGGATTTCAACCATGGG	pGAY1
SB2762	5′ GATCCCCATGGTTGAAATCCCCTCAAAAACCCGATATAATGGGTTTAGT AGCTAAGCTAAAGGGGAATGAACTTCG	pGAY2
SB2763	5′ AATTCGAAGTTCATTCCCCTTTAGCTTAGCTCTAAACCCATTATATCG GGTTTTTGAGGGGATTTCAACCATGGG	pGAY2
SB2764	5′ GATCCCCATGGTTGAAATCCCCTCAAAAACCCGATATAATGGGTTT AATTGAGGTGAGATGATTGGAGAAAG	pGAY3
SB2765	5′ AATTCTTTCTCCAATCATCTCACCTCAATTAAACCCATTATATCGGGTTT TTGAGGGGATTTCAACCATGGG	pGAY3
**Primers for the construction of overexpression plasmids**		
SB2654	5′ ATCAAGCTTCCTAAAGAATGGGACAAGCAA	pUCBAS
SB2655	5′ ATCGGATCCGCCTGTCCTTCGCTGCTG	pUCBAS
SB2690	5′ ATGAATTATGTTATAATAGAGTCAGAGCTAAGCTAAAGGGGAATG	pUCBYJ
SB2691	5′ TATGGTAGGAACTAAAGGAGA	pUCBYJ
SB2635	5′ ATCGGATCCAAGGACAGGCGCTAATCAAC	pUCBYU
SB2636	5′ ATCAAGCTTGCAAAAAAAGACGTTTGCCTAAGGCAAACGTCTTTTTATT TAATTATCCTTGTGTTGCTGCATG	pUCBYU
SB2660	5′ ATCGGATCCATAGGAAAGGAGGTGTACATATAACTTGAGAAAATGGG TATC	pAPYT3
SB2661	5′ ATCGAATTCGCAAAAAAAGACGTTTGCCTAAGGCAAACGTCTTTTCTCTC ATCGGCGTATACGTTGGCG	pAPYT3
SB2722	5′ ATCAAGCTTGAGCTAAGCTAAAGGGGAATG	pDRYJ
SB2723	5′ ATCGCATGCGCAAAAAAAGACGTTTGCCTAAGGCAAACGTCTTTTT GATTAGTGTGACTTGTTTCTTAG	pDRYJ
